# Rapid Biochemical Mixture Screening by Three-Dimensional Patterned Multifunctional Substrate with Ultra-Thin Layer Chromatography (UTLC) and Surface Enhanced Raman Scattering (SERS)

**DOI:** 10.1038/s41598-017-18967-7

**Published:** 2018-01-11

**Authors:** Bi-Shen Lee, Pi-Chen Lin, Ding-Zheng Lin, Ta-Jen Yen

**Affiliations:** 1 Department of Material Science and Engineering, National Tsing Hua University, Hsinchu, 30013 Taiwan; 2 Department of Material and Chemical Research Laboratories, Industrial Technology and Research Institute (ITRI), Hsinchu, Taiwan

## Abstract

We present a three-dimensional patterned (3DP) multifunctional substrate with the functions of ultra-thin layer chromatography (UTLC) and surface enhanced Raman scattering (SERS), which simultaneously enables mixture separation, target localization and label-free detection. This multifunctional substrate is comprised of a 3DP silicon nanowires array (3DP-SiNWA), decorated with silver nano-dendrites (AgNDs) atop. The 3DP-SiNWA is fabricated by a facile photolithographic process and low-cost metal assisted chemical etching (MaCE) process. Then, the AgNDs are decorated onto 3DP-SiNWA by a wet chemical reduction process, obtaining 3DP-AgNDs@SiNWA multifunctional substrates. With various patterns designed on the substrates, the signal intensity could be maximized by the excellent confinement and concentrated effects of patterns. By using this 3DP-AgNDs@SiNWA substrate to scrutinize the mixture of two visible dyes, the individual target could be recognized and further boosted the Raman signal of target 15.42 times comparing to the un-patterned AgNDs@SiNWA substrate. Therefore, such a three-dimensional patterned multifunctional substrate empowers rapid mixture screening, and can be readily employed in practical applications for biochemical assays, food safety and other fields.

## Introduction

In practical applications of biological or chemical rapid detections, most samples are complicated mixtures, and thus, one simultaneously requires two functions of excellent sensitivity and selectivity. Among several detection methods, Raman scattering is a promising candidate for rapid detection because it does not demand labeling process but directly reveals the fingerprint spectra of targeting molecules. In addition, in contrast to conventional infrared absorption techniques, Raman signal is exempt from water interference, giving it permission for wet samples^1,2^. However, the relatively weak Raman scattering cross section will limit its application in the real world conditions. Thus, a phenomenon termed surface enhanced Raman scattering (SERS) proposed by Fleischmann *et al*. in 1970s. With SERS effect, the signal intensity of pyridine molecules attached on the silver electrode could be enhanced by several orders of magnitude^3^. Also, by means of SERS technique, the detections of single molecule^4–6^ were also successfully demonstrated. The dominant mechanism for SERS technique is the excitation of localized surface plasmons^7^ and some relevant works related to Fano resonances and SERS also have been proposed^8–10^.

Nevertheless, the real detected sample is complicated in most cases. If the sensing target is not purified from the complex mixtures, the strong background noise will severely defeat the signal from the target molecules such that its characteristic Raman signals could be overwhelmed or interfered. To solve this issue, certain pre-treatment process, such as purification or extraction is adopted to improve the signal to noise ratio during the detection^11,12^. Within several separation or purification processes, one promising route is thin layer chromatography (TLC). TLC is a simple method to separate different components and relocate them, which is usually applied for the separation of dye mixtures. In the TLC development process, there are two phases, one is stationary phase and another is mobile phase. The stationary phase functioned as a robust channel for molecule migration, while the mobile phase acted as a stream to migrate molecules to a certain position along the flowing direction of mobile phase. With an improvement in the stationary phase, the ultra-thin layer chromatography (UTLC) was invented with the thickness of stationary phase less than 10 μm^13^. The advantages of UTLC are the short migration distance and short development time as well as the low-consumption of mobile phase comparing to conventional TLC. As a result, researchers proposed many kinds of fabrication methods of UTLC substrates such as glancing angle deposition (GLAD)^14–16^, atomic layer deposition^17^, electro spinning^18,19^ and so on.

Although UTLC can separate mixtures efficiently, its detection limit remains too low to be employed for rapid biochemical mixture screening. In 1977, the route combining TLC and SERS techniques was proposed by Henzel *et al*.^20^. Recently, more researchers applied this technique to detect different biochemical molecules including artist dyestuffs^21^, dye and toxin mixtures^22–25^, pollutants in water^26^, or chemical reactions^27^. Among these works, Yu *et al*. presented a paper-based SERS substrate by inkjet printing process^25^. By means of cutting paper-based substrate to specific shapes, the chromatography separation and SERS detection could be achieved by the cellulose paper and metal nanoparticles on the paper, respectively. However, for this kind of paper-based substrates, one demands to limit the power of laser used in Raman measurements, otherwise, the paper-based scaffold will be damaged and even burned. As a consequence, to satisfy practical requests for rapid biochemical mixture screening, herein we demonstrate a novel three-dimensional patterned substrate, composed of silver nano-dendrites decorated at three dimensional patterned silicon nanowire arrays (3DP-AgNDs@SiNWA), to enable UTLC and SERS simultaneously. With the precisely designed pattern, the chromatography separation efficiency was improved and stronger SERS signal was obtained.

## Materials and Methods

### Fabrication of UTLC-SERS multifunctional substrate

**Figure 1:**
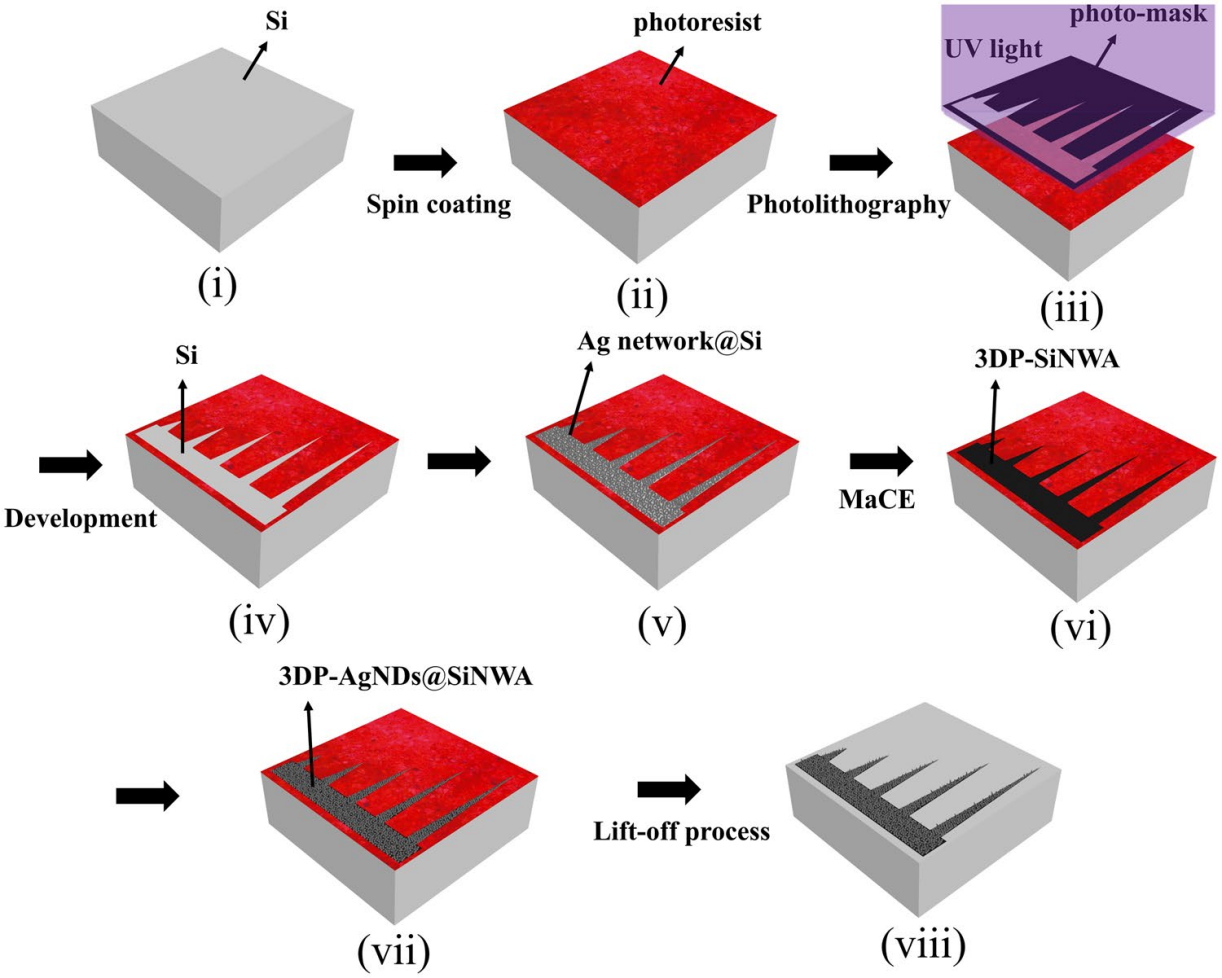
Fabrication flowchart of a UTLC-SERS multifunctional substrate. (**i**) The cut (100) P-type 1.6 × 1.6 cm^2^ silicon chip (gray). (**ii**) Silicon chip with photoresist (red) by spin coating process. (**iii**) UV lithography with a plastic facile mask (patterned black). (**iv**) Patterned region after lift off process. (**v**) Silver network (dark gray) was formed as the catalyst for the following etching process. (**vi**) SiNWA scaffold was obtained after wet-etching process. (**vii**) The fabricated 3DP-AgNDs@SiNWA multifunctional substrate. (**viii**) 3DP-AgNDs@SiNWA multifunctional substrate after removing the residual photoresist.

To grow silicon nanowires, a p-type silicon chip with a size of 1.6 × 1.6 cm^2^ as shown in step (i) of Fig. 1 was first rinsed through acetone, isopropyl alcohol (IPA) and DI water, cleaned in an ultrasonically vibration machine for 10 minutes and dried under nitrogen purge. Next, to pattern the chip, conventional UV photolithography process was then carried out including spin coating a 2-μm-thick photoresist as shown in step (ii) (EPG-516, Everlight Electronics Co., CAS number 108-65-6) with a spin rate of 600 rpm for 5 s followed by 5000 rpm for 30 s, pre-bake process, UV exposure of 80 mJ/cm^2^ by a facile plastic mask as shown in step (iii) and lift off process by 1% KOH solution for 15 s as shown in step (iv) and finally post-bake process. In addition, an anisotropic etching method^28–30^, metal assisted chemical etching (MaCE) was applied with the following steps: 1. Dip the as-prepared Si chips into an electrolyte of hydrogen fluoride (HF) and silver nitrate (AgNO_3_) with concentrations of 4.6 M and 0.44 M, respectively for 10 s to form a silver network as shown in step (v)^31^. 2. Immerse the sample into an etching solution composed of HF and hydrogen peroxide (H_2_O_2_) with concentrations of 4.6 M and 0.44 M, respectively for 10 minutes. Finally, the 3DP-SiNWA with 10 μm depth were obtained as shown in step (vi). Note that the depths of SiNWA can be well controlled by the etching time due to the linear reaction of MaCE^32^.

To achieve a multifunctional UTLC-SERS substrate, electroless chemical deposition method was employed to deposit AgNDs on the surface of 3DP-SiNWA. The sample was dipped into a solution of HF and AgNO_3_ with concentrations of 4.6 M and 0.01 M, respectively, for 120 seconds and the color of substrate would change from black to gray due to stronger reflection from the metallic particles, indicating the formation of 3DP-AgNDs@SiNWA as shown in step (vii). In the final step, the residual photoresist in the un-patterned region was removed by acetone as shown in step (viii). The entire fabrication scheme is depicted in Fig. 1.

### Optimization of SERS effect by different reaction time to form AgNDs

**Figure 2:**
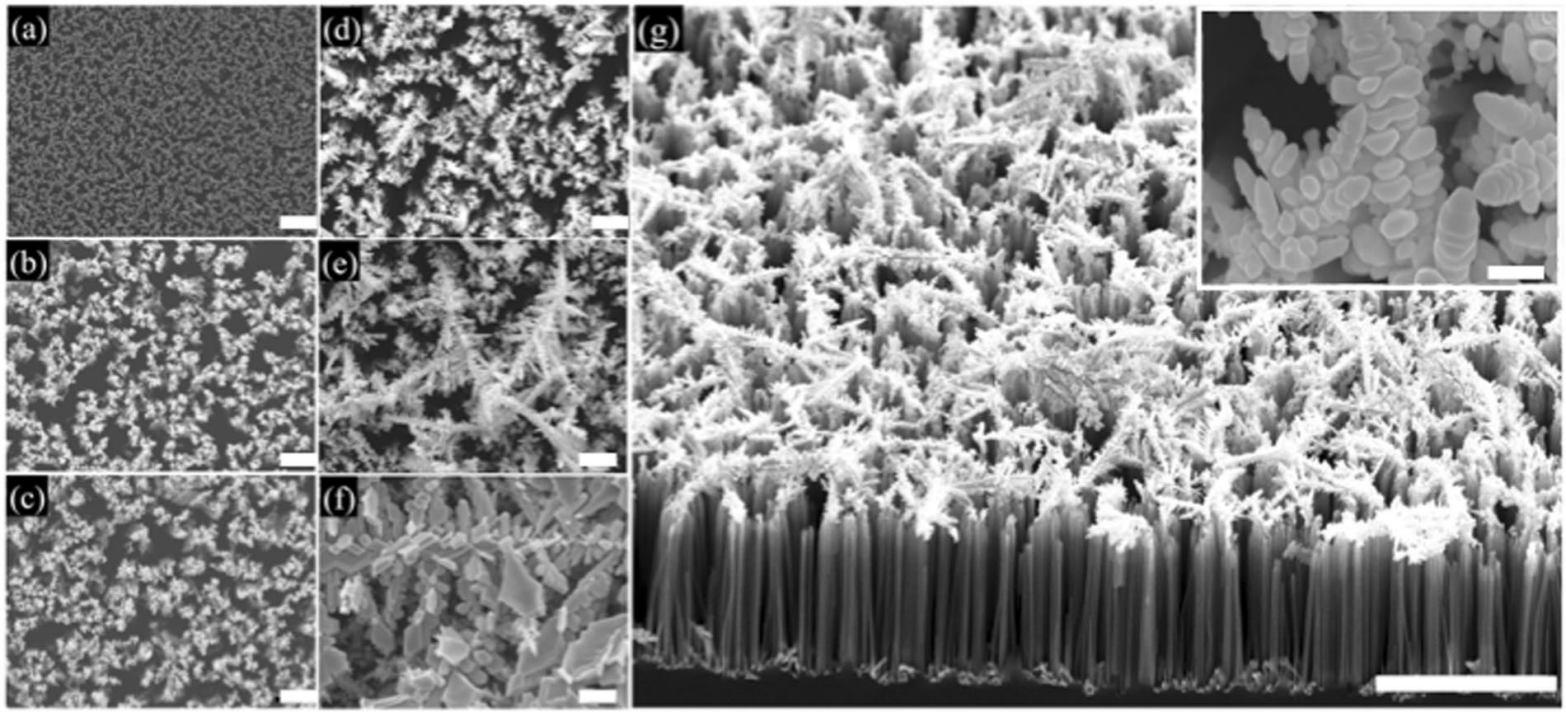
(**a**–**f**) Top view SEM images of AgNDs@SiNWA with different reaction time to form AgNDs (**a**) 0 s (**b**) 30 s (**c**) 60 s (**d**) 90 s (**e**) 120 s (**f**) 180 s. The scale bar represent to 1 μm. (**g**) Cross section SEM image of 3DP-AgNDs@SiNWA with 10 μm depth SiNWA and 120 reaction time to form AgNDs. The inset higher magnitude figure displayed the enlarged AgNDs image (The scale bar represented 10 μm in the lower magnitude SEM figure and represented 200 nm in the inset higher magnitude figure).

**Figure 3:**
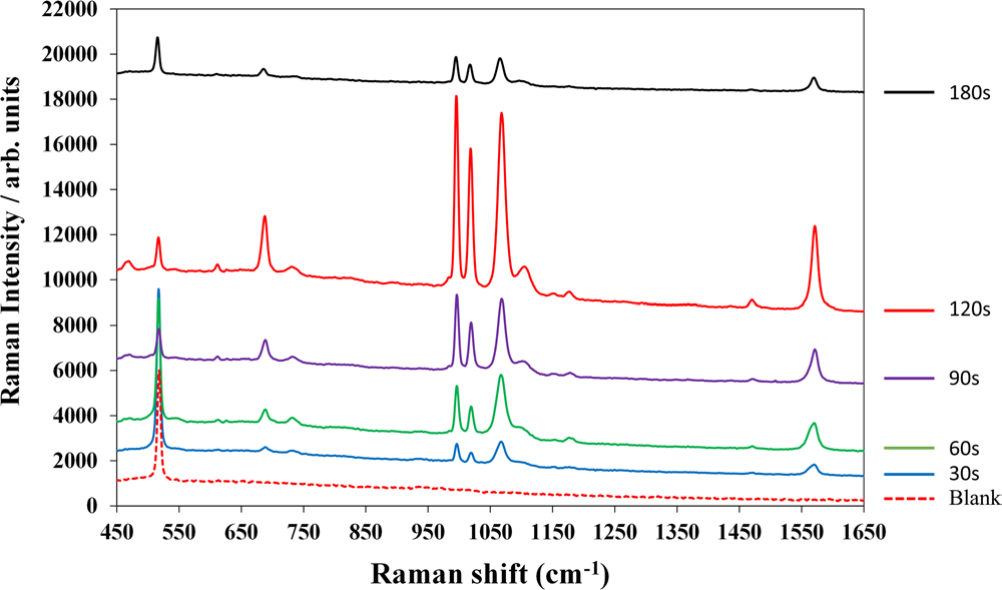
Raman spectra of thiophenol molecules obtained from substrates with different reaction time to form AgNDs. Note that the red dashed line represented the clean background of optimized substrate with 120 seconds reaction time.

After forming the SiNWA scaffold, different reaction time to from AgNDs onto the SiNWA greatly influenced the SERS effect. Therefore, substrates with the same depth of SiNWA and reaction time from 0 to 180 seconds to form AgNDs were chosen as a comparison. Note that the depth of SiNWA was set as 10 μm and the reaction time equal to 0 second represented the pure SiNWA scaffold without silver as shown in Fig. 2(a) (by instrument JSM-7000F from JEOL, Inc.). In the early stage, the high concentration silver salt and reduction agent leaded nucleation-growth forming AgNPs. Small AgNPs formed on the top of SiNWA by the reduction reaction with reaction time equal to 30, 60, and 90 seconds as shown in Fig. 2(b–d), respectively. As the reaction continues, the concentrations of both the silver salt and the reduction agent greatly decreased. Therefore, the growth of silver was mainly driven by the decreased surface energy resulting in the formation of the AgNDs as shown in Fig. 2(e) with reaction time up to 120 seconds. The precisely gap between metals in nanometer scale will greatly enhance the Raman signal intensity^33^. When the reaction time was longer to 180 seconds, the residual micro-scale dendrites would influence the uniformity of SERS signals and might disturb the migration of mobile phase as shown in Fig. 2(f). The cross section SEM image of the optimized substrate with 120 seconds reaction time to form 3DP-AgNDs@SiNWA was shown as Fig. 2(g) (Cross section view of Fig. 2(e)). The inset higher magnitude figure displayed the enlarged AgNDs image, indicating the gap between each silver nanoparticles in the silver dendrites was less than 20 nm. Besides, the corresponding Raman spectra of thiophenol molecules measured by these substrates were shown as Fig. 3.

For the setup of Raman measurement, a 785 nm near infrared laser with power equal to 200 mW was utilized to excite the substrate. The laser beam was focused by objective lens, resulting in a focal spot diameter of 150 μm. Therefore, the illumination intensity was calculated about 0.133 kW/cm^2^ and the laser integration time was set as 1 second for 10 times average. Note that the setups of instruments in Raman measurement are the same in the following sections. For optimizing the substrate, 110 mg thiophenol (99+%, Alfa Aesar Inc., CAS number 108-98-5) was dissolved into 100 ml ethanol to obtain a 10^−2^ M thiophenol solutions. The prepared substrates were then immersed into 10^−2^ M thiophenol solutions for six hours to ensure the specific binding of thiophenol molecules on the SERS substrate. Following the reaction period, samples were removed from ethanolic thiophenol solution, copiously rinsed in ethanol, and dried in nitrogen gas to remove unreacted thiophenol molecules and other solvent. In this process, a self-assembled monolayer of thiophenol was formed on the surface via S-Ag bonds. The characteristic peaks of thiophenol molecule are sited at 999, 1024, 1073 cm^−1^ which represent the in-plane ring-breathing mode, in-plane C-H bend, and in-plane ring-breathing mode coupled with the C-S stretching mode, respectively. Here, we chose the most intensive peak sited at 1073 cm^−1^ to compare the performance between different substrates. Regarding to substrates with 30, 60, and 90 seconds reaction time, few and tiny AgNPs formed on the SiNWA scaffold and leaded to a weaker SERS effect. On the other hand, for the substrate with reaction time longer to 180 seconds, the SERS effect was reduced by over-saturated multilayer micro-scale silver dendrites. Therefore, the reaction time equal to 120 seconds was chosen as a suitable parameter to form optimized UTLC-SERS substrate.

### Design of 3DP-AgNDs@SiNWA UTLC-SERS multifunctional substrate

**Figure 4:**
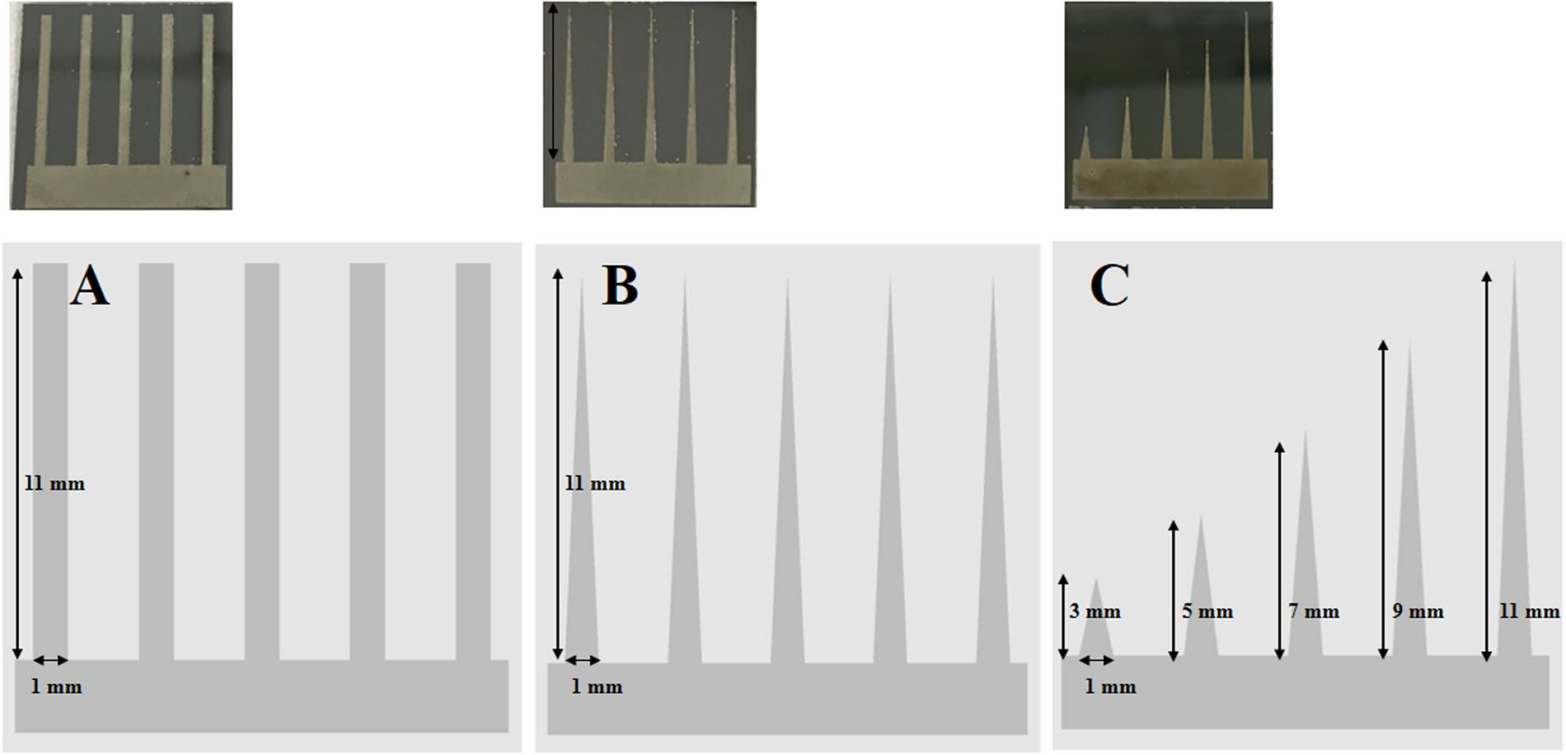
The 3DP-AgNDs@SiNWA UTLC-SERS multifunctional substrate with three different patterns. (**A**) Rectangular shape with same length. (**B**) Tapered shape with same length. (**C**) Tapered shape with various lengths.

To maximize the signal intensity, we designed 3DP-AgNDs@SiNWA UTLC-SERS multifunctional substrate with three different patterns as shown in Fig. 4. Note that the pattern act as channels to guide the analyte migration in the UTLC development process. For pattern A, the designed rectangular channel could confine and direct the migration direction when the analyte molecules moving along the mobile phase. Next, for pattern B, the tapered channel not only confines and directs the migration, but further concentrates the analyte molecules during migration because of shrinking area of the channel. The channel width and length of pattern A and B were equal to 1 mm and 11 mm, respectively. Note that the width of channel was set based on the laser beam spot equals to 150 μm in the following SERS measurement. Finally, for pattern C, various lengths of the tapered channels were designed for the target molecule exactly relocating near the tip end to maximize the concentration effect. The lengths of channels in pattern C were equal to 3, 5, 7, 9, and 11 mm from the left to right ones to meet the requirement of different samples. Benefited from the confinement, concentrate and relocalization effects, we expect that the integrated performance of UTLC separation and SERS detection will be substantially improved, outperform the un-patterned substrate.

### Process flow of UTLC-SERS measurement process

**Figure 5:**
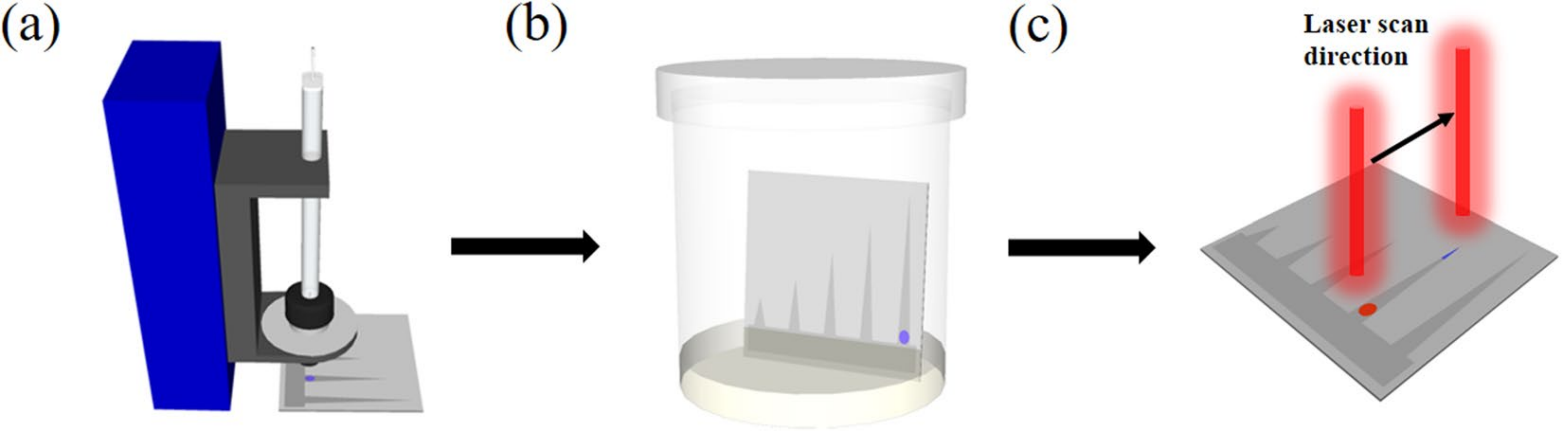
Schematic diagram of UTLC-SERS process. (**a**) Samples were spotted onto UTLC-SERS multifunctional substrate (original spot). (**b**) The UTLC development process with certain amounts of mobile phase in a developing tank. (**c**) Onsite Raman screening measurement along the migration direction of mobile phase with a 1 mm interval after UTLC development process.

To demonstrate the high-performance rapid screening for mixtures by the fabricated 3DP-AgNDs@SiNWA multifunctional substrates, three samples, 10^−4^ M rhodamine 6G (R6G) (Sigma Aldrich Inc., CAS number 989-38-8) solutions, 10^−4^ M methylene blue (MB) (Sigma Aldrich Inc., CAS number 122965-43-9) solutions and their mixtures were chosen. As shown in Fig. 5(a), the samples were spotted on the fabricated 3DP-AgNDs@SiNWA multifunctional substrate by a spotting instrument (Linomat 5 by CAMAG inc.). Then, the substrate with mixtures was transferred into a UTLC development tank with saturated vapor pressure of mobile phase, as shown in Fig. 5(b). Note that the original spotting position cannot be lower than the level height of the mobile phase. Hence, the original spotting position was set as 4 mm away from the bottom side of the substrate and the height of the mobile phase was set as 2 mm away from the bottom side of the substrate. After UTLC development process, the substrate was removed from the development tank and dried under atmosphere. Finally, the superimposed Raman spectra were then obtained with a 1 mm interval along the migration direction from the original spotting position, as shown in Fig. 5(c).

## Results

### Surface property of fabricated UTLC-SERS multifunctional substrate

**Figure 6:**
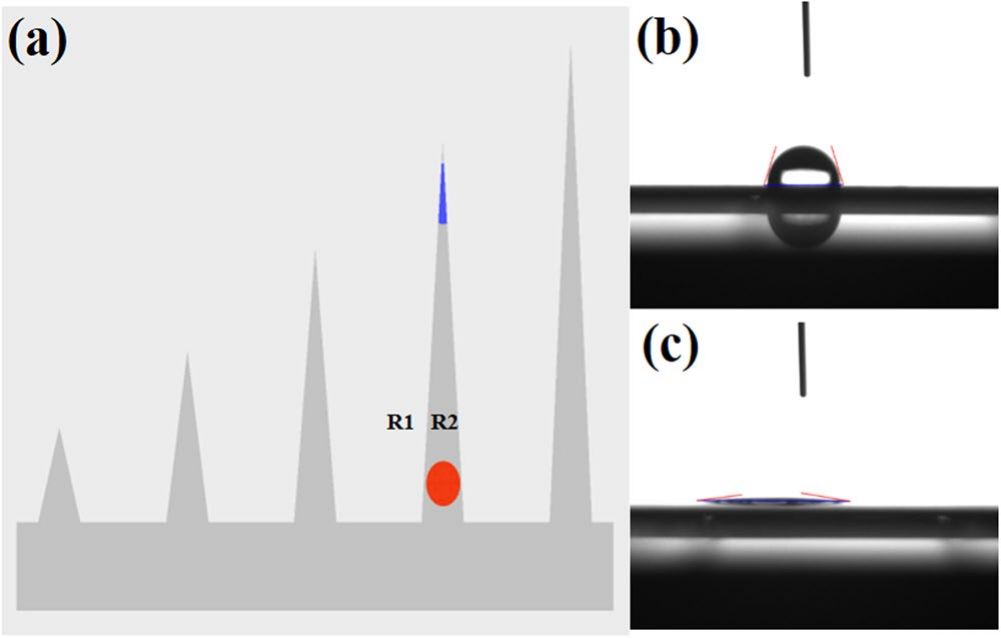
(**a**) R1 and R2 represented the pure silicon region and 3DP-AgNDs@SiNWA region, respectively. (**b**) The corresponding water contact angle (near 70 degrees) of R1. (**c**) The corresponding water contact angle (near 6 degrees) of R2.

The morphology of the fabricated 3DP-AgNDs@SiNWA multifunctional substrates was examined in previous section as shown in Fig. 2(g). The silicon nanowires array (SiNWA) is straight and vertically aligned with the uniform length of 10 μm, which functions excellent UTLC property. This SiNWA is also a scaffold to accommodate the AgNDs atop by immersing the SiNWA into HF and AgNO_3_ solution of 120 seconds. The decorated dense AgNDs facilitate the SERS detection due to the localized surface plasmon resonance (LSPR)^34,35^. In addition, as mentioned earlier, the designed pattern acted as the channel for UTLC development process and possessed the “confinement” effect of molecule migration. Outside the patterned region (flat silicon region), the measured water contact angle was near 70 degrees (i.e., R1 in Fig. 6(a)), as shown in Fig. 6(b). In contrast, the water contact angle turned to be 6 degrees in the patterned region (i.e., R2 in Fig. 6(a)), as shown in Fig. 6(c). This super-hydrophilic property results from the nanostructure of SiNW inside the patterned region^36^. Therefore, such enormous contrast of water contact angles between R1 and R2 gives rise to the well confinement in the patterned channels, leading to excellent UTLC-SERS performance.

## Discussion

### Separation and detection of dye mixtures by 3DP-AgNDs@SiNWA multifunctional substrate

**Figure 7:**
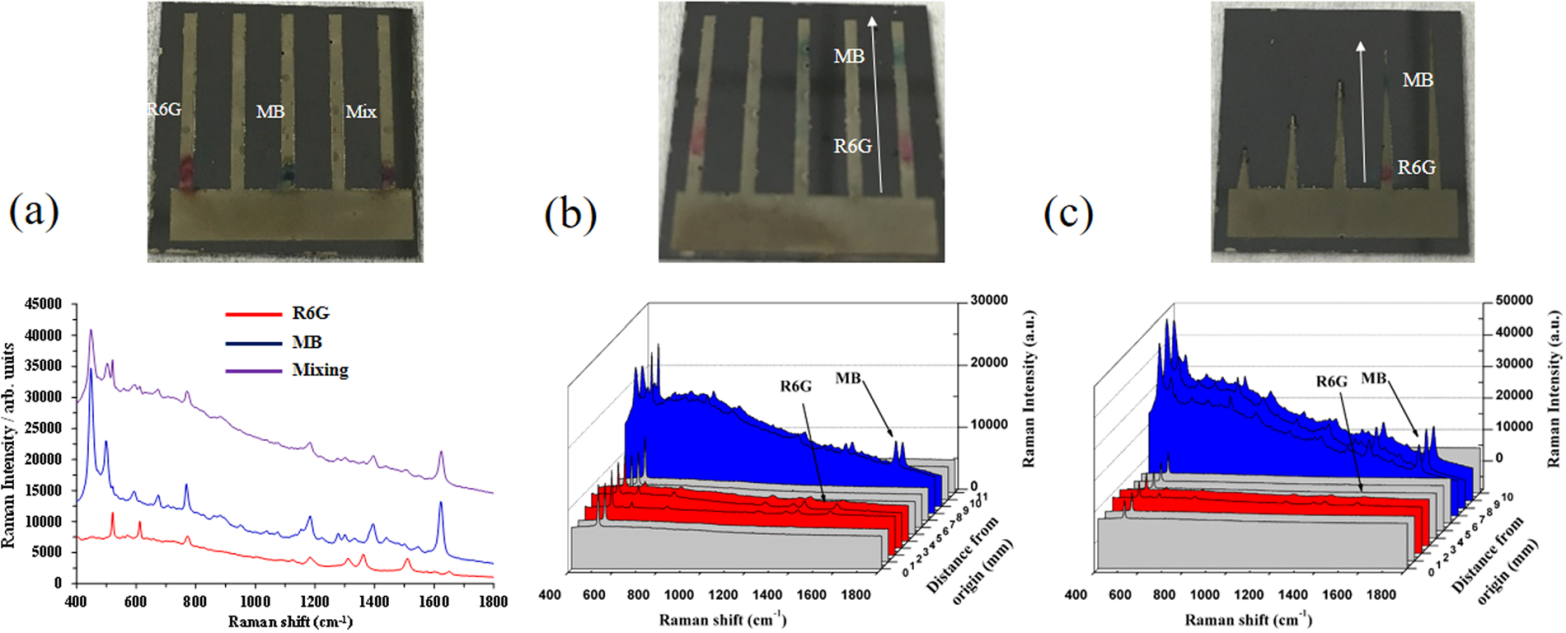
(**a**) The SERS spectra of R6G solution (10^−4^ M), MB solution (10^−4^ M) and their mixtures before UTLC process. (**b**) The superimposed SERS spectra of dye mixtures on the rectangular shape pattern after UTLC development process. (**c**) The superimposed SERS spectra of dye mixtures on the tapered shape pattern with suitable length after UTLC development process. Note that the red and blue region represented the position of R6G and MB respectively.

First of all, three samples, R6G solution (10^−4^ M), MB solution (10^−4^ M) and their mixtures with volume equal to 20 μl were spotted onto fabricated substrate before the UTLC process, and the corresponding SERS spectra were shown in Fig. 7(a). The red line denotes the SERS spectrum for R6G solution, the blue line for MB solution, and the purple line for the mixture of R6G and MB, respectively. It was obvious that the characteristic peaks of R6G (1511 cm^−1^) and MB (1625 cm^−1^) in the mixture were much weaker than those in their one solution. In addition to weaker Raman signals, a more critical problem without the UTLC separation process is the signal overlapping, which makes the detection of individual targets in the mixture impossible. As a result, a UTLC development process becomes a must to achieve excellent sensitivity and selectivity in practical applications. After the UTLC development process, R6G and MB molecules were relocated to specific positions because of different affinities to stationary phase. The ethyl acetate (EA) was applied as the mobile phase in this UTLC development process. As shown in Fig. 7(b), the superimposed Raman spectra obtained with 1 mm interval along the migration direction indicated that the migration distances for R6G and MB were about 3 mm and 8 mm, successfully demonstrating the separation of dye mixtures. If we further applied the substrate with suitable length of tapered shape pattern to separate and detect the R6G and MB individually, the signal intensity could be further stronger comparing to the previous two cases as shown in Fig. 7(c).

### Comparison between un-patterned AgNDs@SiNWA and different kinds of 3DP-AgNDs@SiNWA UTLC-SERS multifunctional substrate

**Figure 8:**
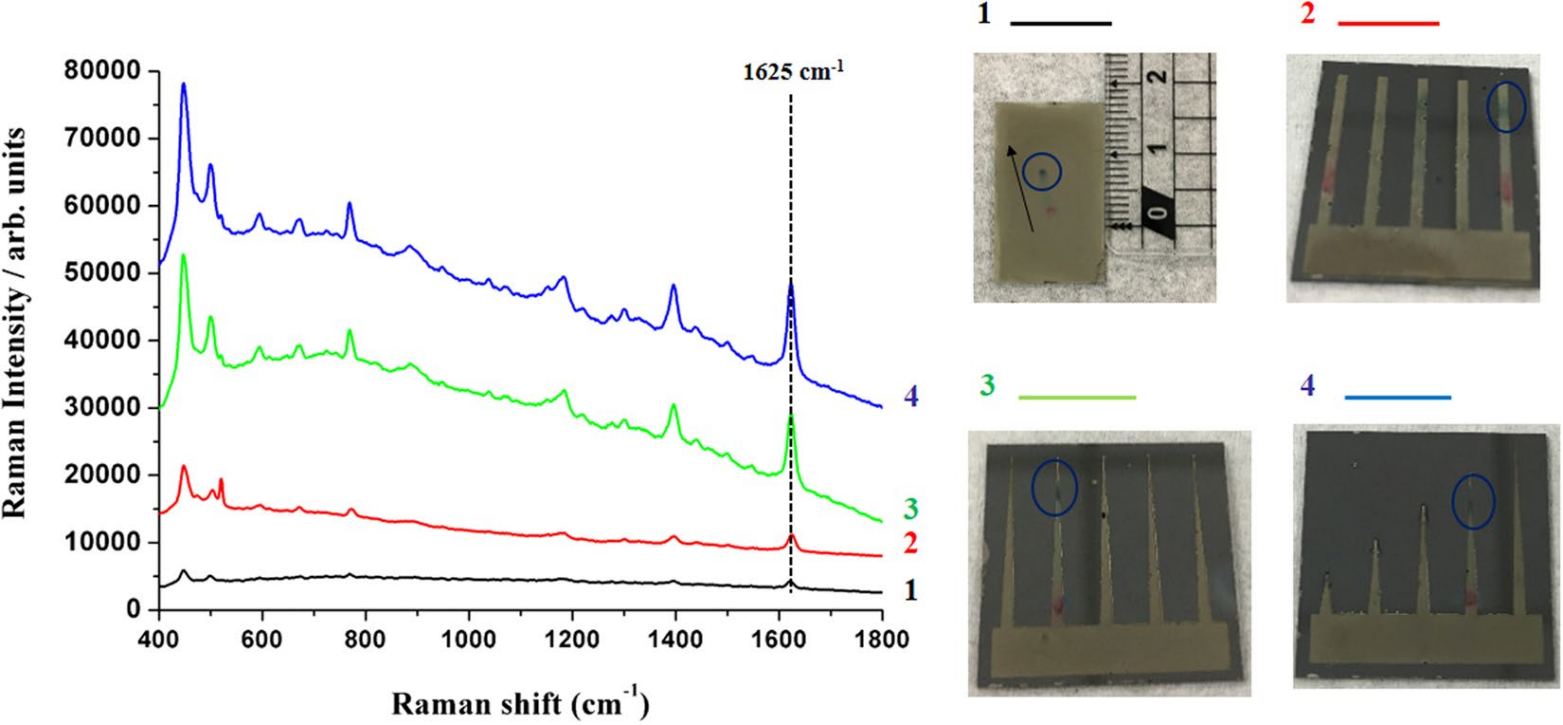
The Raman spectra of MB after UTLC-SERS process by different kinds of multifunctional substrates. The peak sited at 1625 cm^−1^ is chosen to compare the peak intensity of MB. Note that all curves were tracked at the 8 mm position after the UTLC separation process and the concentration of R6G and MB is both equal to 10^−4^ M.

In previous section, the function of designed patterns was successfully verified by detecting the R6G and MB from their mixtures. Finally, we displayed the comparison of Raman signal intensity from the target by different kinds of UTLC-SERS multifunctional substrates. Three 3DP-AgNDs@SiNWA (rectangular, tapered, tapered with various lengths) and the un-patterned one were chosen for a comparison based on the peak intensity of MB (at 1625cm^−1^) tracked at the same position of 8 mm (the position with strongest signal intensity) after the UTLC-SERS process. As shown in in Fig. 8, the curve 1 (black color) represented the signal from the un-patterned AgNDs@SiNWA multifunctional substrate, as a control group in this comparison. Under this condition, the migration of dye molecules showed a slightly drift along the mobile phase migration direction due to the small defects inside the SiNWA after the UTLC separation process. Owing to the randomly drift of molecules, the peak intensity of MB at 1625 cm^−1^ was merely about 780 counts/s. With explicitly designed rectangular pattern of acting as a “confinement” channel during UTLC separation process, the signal intensity of MB increased to 2211 counts/s by the rectangle pattern, near three times stronger than the un-patterned case, as shown by the curve 2 (red color) in Fig. 8. Then, with the tapered pattern, which showed a further “concentrated” effect to localize the analyte to the position near the tip, the peak intensity of MB roared to 9273 counts/s indicated by the curve 3 (green color) in Fig. 8. Eventually, with the designed sequential tapered patterns, the signal intensity of MB was maximized to 12029 counts/s by the both confinement and concentrated effect, as demonstrated by the curve 4 (blue color) in Fig. 8. The second longest channel with length equals to 9 mm was chosen because of the suitable length to relocate the MB molecule near to the tip-end. In the end, the signal intensity of MB was amplified from 780 to 12029 counts/s (15.42 times) by the optimized 3DP-AgNDs@SiNWA substrate comparing to the un-patterned one.

In conclusion, we demonstrated facile photolithographic and low-cost wet chemical processes to fabricate the multifunctional substrate of 3D patterned silver nano-dendrites decorated at silicon nanowires array (3DP-AgNDs@SiNWA). The 3DP-SiNWA provides an effective platform for mixture separation and target localization; and the AgNDs can dramatically intensify the Raman signals of the target. By means of this 3DP-AgNDs@SiNWA substrate, we successfully integrated the excellent multifunction of ultra-thin layer liquid chromatography (UTLC) and surface enhanced Raman scattering (SERS), to effectively analyze the dye mixtures. We further optimized this multifunctional substrate, by introducing various tapered patterns on the substrates. Due to their super-hydrophilic property, these patterned AgNDs@SiNWA enable much more efficient confinement and concentration effects, leading to strong localization of individual targets from the mixtures. The measured Raman signal of MB from the mixture showed a 15.42-fold enhancement of magnitude, in contrast to the case of the un-patterned one. As a result, this three-dimensional patterned multifunctional substrate successfully displayed the remarkable functions of UTLC and SERS for rapid mixture screening, promising the practical applications in biochemical assays, food safety and other fields.
